# Sulforaphane response on aluminum-induced oxidative stress, alterations in sperm characterization and testicular histomorphometry in Wistar rats

**DOI:** 10.18502/ijrm.v13i8.7503

**Published:** 2020-08-19

**Authors:** Babatunde Ogunlade, Sunday Adelakun, Kingsley Iteire

**Affiliations:** ^1^Department of Human Anatomy, Federal University of Technology, Akure, Ondo State, Nigeria.; ^2^Department of Human Anatomy, University of Medical Sciences, Ondo city, Ondo State, Nigeria.

**Keywords:** Sulforaphane, Aluminum trichloride, Oxidative stress, Testis, Histology.

## Abstract

**Background:**

The exposure of male individual to environmental toxicant is regarded as a channel that results in reduced sperm counts and infertility.

**Objective:**

This study investigated the ameliorative response of Sulforaphane (SFN) on Aluminum trichloride (AlCl${}_{3}$) induced testicular toxicity in adult male Wistar rats.

**Materials and Methods:**

A total of 32 adult male Wistar rats (180-200 gm between 8-10 wk) were divided into four groups (n = 8/each). Group A) received distilled water orally as placebo; Group B) received 100 mg/kgbw AlCl${}_{3}$ only orally; Group C) received 100 mg/kgbw AlCl${}_{3}$ and 100 mg/kgbw SFN orally; and Group D) received 100 mg/kgbw SFN only orally. After 28 days of experiment, animals underwent cervical dislocation, blood serum was obtained for analysis, and testes were harvested for biochemical assays, histology, hormonal profile, and sperm characterization.

**Results:**

The sperm parameters showed a significant difference within the AlCl${}_{3}$ only group compared with the control and SFN only groups (p = 0.02). However, AlCl${}_{3}$ and SFN co-treatment showed improvement in the motility, viability, and sperm count compared with the AlCl${}_{3}$ only group (p = 0.02). Furthermore, there was a significant decline in the levels of hormones profile and antioxidant status in AlCl${}_{3}$ only group compared to the control and SFN only (p = 0.02). The testicular histoarchitecture of the AlCl${}_{3}$ only group showed shrinkage of seminiferous tubules, spermatogenesis disruption, and empty lumen compared to the control and SFN only groups.

**Conclusion:**

The present study revealed the ameliorative response of SFN on AlCl${}_{3}$-induced testicular toxicity on serum hormone profiles, antioxidant status, lipid peroxidation, and histomorphometric analysis through oxidative stress.

## 1. Introduction

The exposure of male individual to environmental toxicant is regarded as a channel that results in reduced sperm counts and infertility (1, 2). Aluminum is considered as the most common metallic element detectable in natural waters, animal, and plant tissues (3) leading to a significant upsurge in both gastrointestinal absorption and urinary elimination of aluminum in exposed individuals (4). The affinity of aluminum to other elements stimulates free radical-mediated reproductive cytotoxicity causing impairment of testicular tissues to both humans and animals (5). Aluminum compounds are widely used as by-products for the manufacture of several household cooking utensils andpharmaceutical drugs (such as antacids, vaccines, anti-diarrhea drugs, phosphate binders, and injections of allergy immunotherapy) (6). Increase in the level of exposure to aluminum-containing products will boost the concentration of this metallic element in different organs thereby causing harmful effects to the well-being of humans (7).

In addition, elevated concentrations of Aluminum in human sperm and seminal plasma were observed to decrease sperm viability and motility (8). Testicular Aluminum accumulations cause spermatocyte necrosis and trigger other reproductive toxicity through several mechanisms such as oxidative stress, which ultimately interferes with spermatogenesis and steroidogenesis, bloodtestis barrier, and endocrine disruption (9). The application of nutritional antioxidant supplements has increased over the years to tackle oxidative stress-induced tissue damage since they act as defense regulators and scavengers of reactive oxygen species. Sulforaphane (SFN) is the most active natural products found in crucifers such as broccoli sprout, cabbage, and kale with the potential of lowering the risk of cancer, oxidative stress-induced tissue injury, and age-related diseases (10). SFN possess antiproliferative activities and can effectively halt the initiation and progression of chemically induced tissue damage in animals (11). In addition, SFN has been suggested to have antidiabetic properties for normalizing changes in blood glucose and insulin sensitivity (12-14), and is used in cardiovascular and antihypertensive protection (15, 16). It has been reported that SFN can promote elimination and detoxification of aflatoxin (17), acetaldehyde (18), methylmercury (19), acrolein (20), benzene, crotonaldehyde (21), and free radicals (22) through the Nrf2-mediated mechanism. Furthermore, some clinical studies have demonstrated the effectiveness of SFN supplements in the prevention and/or improvement of skin erythema (23), autism (24), insulin resistance (13), Helicobacter pylori-infection (25), and liver abnormality (26). SNF also has the ability to cause programmed cell death(apoptosis) and cell cycle arrest linked to their ability to regulate several proteins such as Bcl-2 and Bax family proteins, caspases, p21, cyclins, and cyclin-dependent kinases (27).

This study was therefore designed to investigate the ameliorative response of SFN on the histomorphometric and enzymatic antioxidants on Aluminum chloride (AlCl${}_{3}$)-induced testicular toxicity of adult Wistar rats.

## 2. Materials and Methods

### Chemicals 

AlCl${}_{3}$ and SFN were procured from Sigma Company (St. Louis, MO, USA). All other chemicals used in the study were of analytical-reagent grade.

### Animals

In this prospective cohort study, a total of 32 adult male Wistar rats, weighing 180-200 gr and aged 8-10 wk (Rattus norvegicus) were obtained from the animal house, Department of Human Anatomy, Ladoke Akintola University of Technology, Ogbomosho. The rats were collected in an isolated cages in the experimental house of the Department of Human Anatomy, Federal University of Technology, Akure. They were maintained under constant 12 hr light/dark cycle.

### Experimental protocol

The rats were divided into four groups (n = 8/each) Group A) represent control and received water as placebo. Group B) were administered (orally) with 100 mg/kgbw AlCl${}_{3}$ only (in 0.5 ml of distilled water). Group C) were administered (orally) with 100 mg/kgbw AlCl${}_{3}$ (in 0.5 ml of distilled water) and 100 mg/kgbw SFN (in 0.5 ml of distilled water), and Group D) was administered (orally) with 100 mg/kgbw SFN only (in 0.5 ml of distilled water). The experiment lasted for 28 days after which the animals were sacrificed.

All animals were observed for any behavioral anomalies, illness, and physical anomalies. The experimental procedures were conducted in accordance with the provided recommendations in the “Guide for the Care and Use of Laboratory Animals” prepared by the National Academy of Sciences. The rats were fed with standard rat chow and drinking water was supplied ad libitum. The weight of the animals was recorded at procurement, during acclimatization, at commencement of the experiment, and weekly throughout the experimental period using a CAMRY electronic scale (EK5055, Indian).

### Surgical procedure

“After the last administration, the rats were administered intraperitoneal pentobarbital sodium (40 mg/kg) and their abdominal region was opened and the testes of all the animals were immediately removed. The testicular weight of each rat were recorded. The rats were decapitated and blood samples were collected for analysis. The blood samples were centrifuged at 4°C for 10 min at 250× gr and the serum obtained was stored at 20°C until assayed. The harvested testis specimens were fixed in Bouin's fluid for histological analysis” (28).

### Epididymis sperm count, viability, and motility

“The spermatozoa from the cauda epididymis were obtained by cutting into 2 ml of medium (Hams F10) containing 0.5% bovine serum albumin (29). After 5 min of incubation at 37°C (with 5% CO${}_{2}$), the cauda epididymis sperm reserves were determined using a hemocytometer”. Sperm motility, viability (live spermatozoa/death spermatozoa ratio), and morphology (percentage normal spermatozoa, abnormal head defect, and abnormal tail defect) were analyzed with a microscope (Leica DM750) and reported as the mean percentage of motile sperm according to the method developed by the World Health Organization (30).

### Biochemical estimations

The level of lipid peroxidation products were estimated in accordance with the method published by Adelakun and co-workers. (31). Nonenzymatic antioxidants such as reduced glutathione (GSH) and catalase (CAT) were estimated as described by Adelakun and co-workers. The SOD activity in the testes was also determined according to the method described by Adelakun and co-workers (31).

### Hormone determination

“The hormonal levels of testosterone (TT), follicule-Stimulating Hormone (FSH), and Leutenizing Hormone (LH) were measured using available immunoassay (ELISA) kits (Randox Laboratories Ltd, Admore Diamond Road, Crumlin, Co., Antrim, United Kingdom, Qt94QY) according to manufacturer's instructions”.

### Testicular histology preparation

“The testes of the rats were harvested and fixed in Bouin's fluid for 24 hr before being transferred to 70% alcohol for dehydration. The tissues passed through 90% and absolute alcohol and xylene for different durations before being transferred into molten paraffin wax for 1 hr each in an oven at 65°C for infiltration. The tissues were embedded and serial sections cut on a rotary microtome set at 5 microns were performed. The tissues were picked up with albumenized slides and allowed to dry on hot plates for 2 min. The slides were dewaxed with xylene and passed through absolute alcohol (two changes), 70% alcohol, 50% alcohol, (in that order), and then in water for 5 min. The slides were then stained with Hematoxylin and Eosin, mounted in DPX, and photomicrographs were taken at a magnification of 100 × on a Leica DM750 microscope” (31).

### Morphometric studies

“Morphometric studies were carried out with modification of Akang and co-workers (32). Briefly, four sections per testis and six microscope fields per section were randomly chosen for analysis. Fields were sampled as images captured on a Leica DM750 bright field microscope (Germany) via LAZ software. Volume densities of testicular ingredients were determined by randomly superimposing a transparent grid comprising 35 test points arranged in a quadratic array. Test points falling on a given testis and its ingredients were summed over all fields from all sections. The total number of points hitting on a given ingredient (lumen (EL), epithelium (EE), interstitium (EI)), divided by the total number of points hitting on the testis sections (ET) multiplied by 100 provided an unbiased estimate of its percentage volume density/volume fraction. The estimation of the volumes of seminiferous tubule EE (seminiferous EE) and EI in the testes was done in accordance with Howard & Reed (33) and Baines and co-workers” (34).

### Quantitative evaluation of germ cells 

“This was carried out according to the method described by Adelakun and co-workers (31). Briefly, quantitative evaluation of spermatogonia, preleptotene, pachytene spermatocytes, and round spermatid cells was performed using 50 round tubules per group selected in stage VII/VIII of the seminiferous tubule cycle at × 400. The diameters of nuclei of various germ cell types were measured by means of an ocular micrometer and a correction factor was used to obtain the actual numerical density of germ cells” (31).

### Ethical consideration

All animal handling procedure and research activities was approved by the Ethics Committee of the College of Medicine, University of Lagos, Nigeria (CM/ HREC/07/19/120).

### Statistical analysis

Where applicable, data obtained were analyzed statistically using one-way ANOVA, followed by Dunnett's comparison test. Data were expressed as Mean ± SEM. The level of significance was at p < 0.05. Data were analyzed using GraphPad Prism 5 Windows (GraphPad Software, San Diego, California, USA).

## 3. Results

### Testes and body weight

In addition, there was a significant decline in the relative weight of the testes in the rat that received AlCl${}_{3}$ compared with the control (p = 0.02; Table I). However, the co-administration of SFN and AlCl${}_{3}$ showed a recovery in the relative testicular weight and was not statistically different compared with the control.

There was a significant decrease in the body weight in the rats administered with AlCl${}_{3}$ compared with the control (p = 0.02; Table I). However, there were no significant differences in body weight in the group administered with SFN only and combined administration of SFN and AlCl${}_{3}$ compared with the control.

### Effect of AlCl${}_{3}$ on sperm parameters

**Figure 1:**
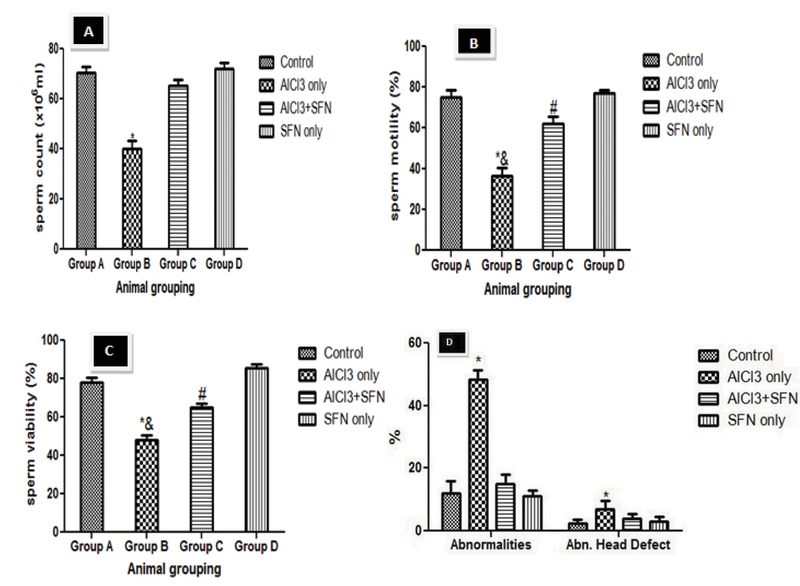
Effect of SFN on sperm count, motility, viability, and sperm morphology on AlCl${}_{3}$ in normal and experimental rats (n = 8). *P < 0.05 compared with the control; &P < 0.02 compared with the SFN + AlCl${}_{3}$ group; #P < 0.05 compared with the control and SFN only group. Values expressed in Mean ± SEM.

The spermatozoa concentration showed a significant difference within the AlCl${}_{3}$ only group compared with the control (p = 0.02; Figure 1A). The co-administration of AlCl${}_{3}$ and SFN group showed increase in sperm count compared with the AlCl${}_{3}$ only group. However, the sperm count of the co-administration of AlCl${}_{3}$ and SFN was significantly lower compared with the control and SFN only group but was not statistically significant.

In addition, there was a significant decrease in sperm motility in the group administered with AlCl${}_{3}$ only compared with the control (p = 0.02; Figure 1B). The group administered with a combination of AlCl${}_{3}$ and SFN showed improvement in the motility of the spermatozoa compared with the AlCl${}_{3}$ only group (p = 0.03; Figure 1B). However, there was no significant difference between the control and SFN only group.

Furthermore, the spermatozoa viability was significantly decreased after AlCl${}_{3}$ administration compared with the control (p = 0.02; Figure 1C). However, the viability of the spermatozoa in the SFN + AlCl${}_{3}$ group showed significant difference compared to the control and SFN only group (p = 0.03; Figure 1C).

The AlCl${}_{3}$ only group had significantly (p = 0.02) higher sperm head defects compared to the control (Figure 1D). However, there was no significant difference in the abnormal head defeat in the groups that received SFN only and a combination of AlCl${}_{3}$ and SFN compared with the control. Furthermore, the AlCl${}_{3}$ only group showed a significantly higher percentage of sperm abnormalities compared to the control (p = 0.02; Figure 1D). The percentage level of sperm abnormalities was drastically reduced in the combined administration of SFN and AlCl${}_{3}$, which was not statistically significant compared to the SFN only and control groups (Figure 1D).

### Serum TT, FSH, and LH 

**Figure 2:**
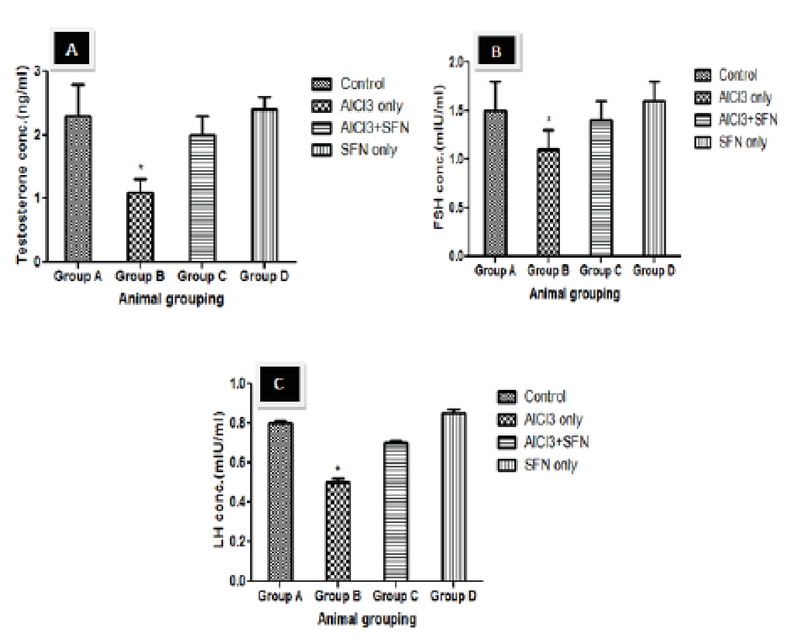
Effect of SFN on serum level of testosterone concentration, FSH, and LH on AlCl${}_{3}$ in normal and experimental rats (n = 8). *P < 0.03 compared with the control. Values expressed in Mean ± SEM.

There was a significant decline in the levels of serum TT, FSH, and LH in rats treated with AlCl${}_{3}$ only compared to the control (p = 0.02; Figure 2 A-C). However, the levels of serum TT, FSH, and LH were significantly improved in the group administered with a combination of SFN and AlCl${}_{3}$ compared with AlCl${}_{3}$ only group. Although, in comparison to control rats and SFN only groups, the recovery in hormonal level was partial and less but it was not statistically significant.

### Lipid peroxidation and antioxidant status

**Figure 3:**
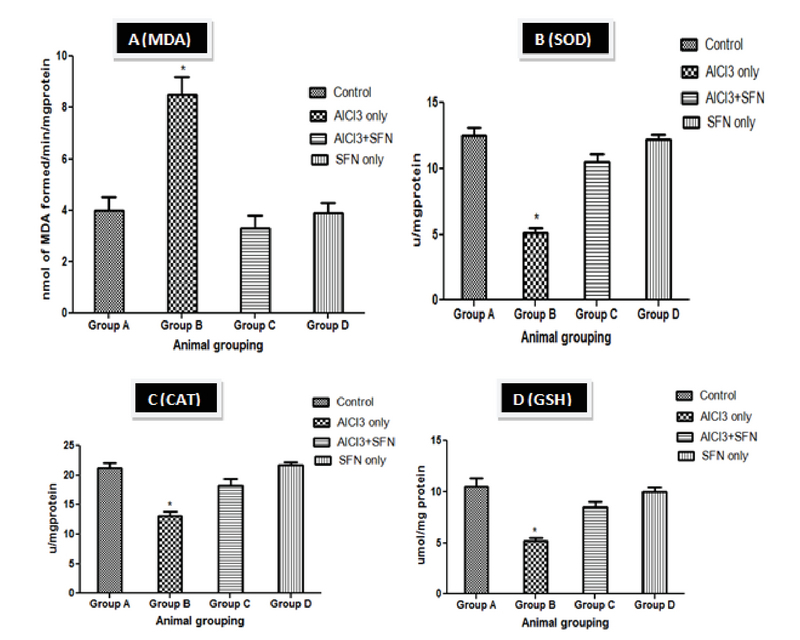
Effect of SFN on Lipid peroxidation and antioxidant status on AlCl${}_{3}$ in normal and experimental rats (n = 8). *P < 0.05 compared with the control. Values expressed in Mean ± SEM.

The rats administered with AlCl${}_{3}$ only showed a significant increase in MDA levels and corresponding decrease in SOD, CAT, and GSH levels compared with the control (p = 0.03; Figure 3 A-D). However, treated group that received a combined administration of SFN and AlCl${}_{3}$ showed a significant improvement of Lipid peroxidation and antioxidant status compared with AlCl${}_{3}$ only group but it was not statistically significant compared with the SFN only and control groups.

### Testicular histology

**Figure 4:**
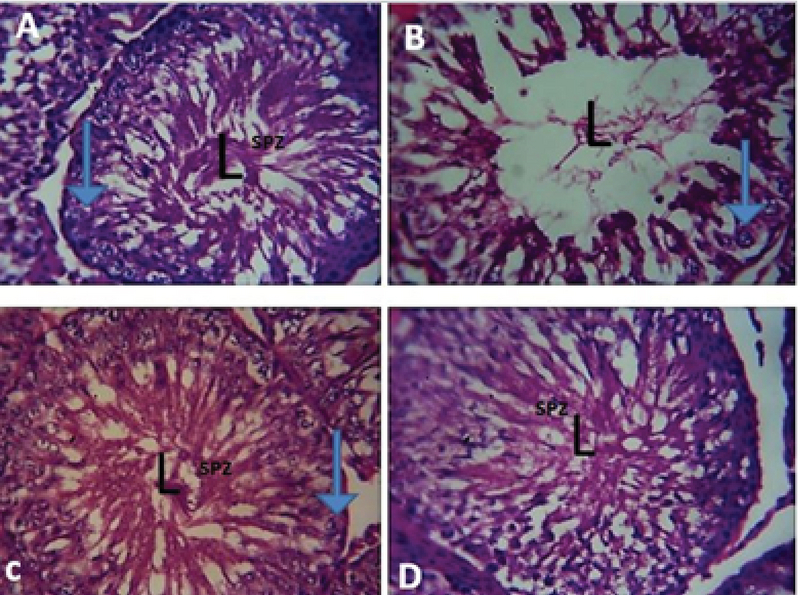
Representative photomicrograph of the effect of SFN on AlCl${}_{3}$-induced testicular toxicity in normal and treated rats. (A) Testicular photomicrograph section of control rat showing no pathological changes in the lumen (L) of the seminiferous tubules, spermatozoa (SPZ), and primary spermatocyte (arrow). (B) Testicular photomicrograph section of AlCl${}_{3}$ only group showing distinctive shrinkage of seminiferous tubules, hypocellularity due to degeneration of germ cells, disruption of spermatogenesis, and empty lumen (L). (C) Testicular photomicrograph section of SFN and AlCl${}_{3}$-treated group showing restored lumen (L) with visible spermatozoa (SPZ) and abundant sperm cell (arrow). (D) Testicular photomicrograph section of SFN only showing normal testicular structure with intact spermatozoa (SPZ) within the lumen (L) (H&E; x400).

The testicular histoarchitecture of the AlCl${}_{3}$ only group showed necrosis and degeneration with decrease in germinal EE thickness and reduction in the diameter of the seminiferous tubules when compared with the control. In addition, AlCl${}_{3}$ caused distortion in the seminiferous tubules with loss of normal distribution of epithelial lining and vacuolar cytoplasm compared with the control. However, testicular photomicrograph of the control section had similar characteristics with the SFN only group showing oval or circular presentation with distinctive stratified seminiferous EE whose EL possesses spermatogenic cells and prominent Leydig cells. The testicular section of the group administered with both SFN and AlCl${}_{3}$ showed restored microarchitecture of the testicular morphology showing mild distortion of the tubular architecture and disorganization of the spermatogenic cells in seminiferous tubules (Figure 4 A-D).

### Stereological analysis

**Figure 5:**
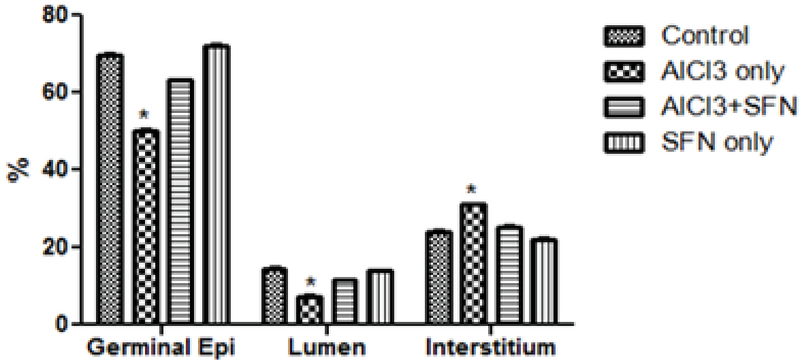
Effect of SFN on morphometric analysis on AlCl${}_{3}$ in normal and experimental rats (n = 8 *P < 0.05 compared with the control. Values expressed in Mean ± SEM.

The volume density of the germinal of the AlCl${}_{3}$ only group showed a significant decrease compared with the control (p = 0.02; Figure 5). However, there was no significant difference in the volume of the germinal EE after the administration of SFN and AlCl${}_{3}$ compared with the SFN only and control groups, respectively. Furthermore, the EL density significantly decreased in the AlCl${}_{3}$ only group compared to the control (p = 0.02; Figure 5), while the combination of SFN and AlCl${}_{3}$ showed no significant difference in the EL density compared to the SFN and control groups.

Concerning the EI, the AlCl${}_{3}$ only group showed a significant increase compared to the control (p = 0.02; Figure 5), while a corresponding decrease was observed in the combined SFN and AlCl${}_{3}$ group but it was not statistically significant compared to the SFN and control groups, respectively.

**Figure 6:**
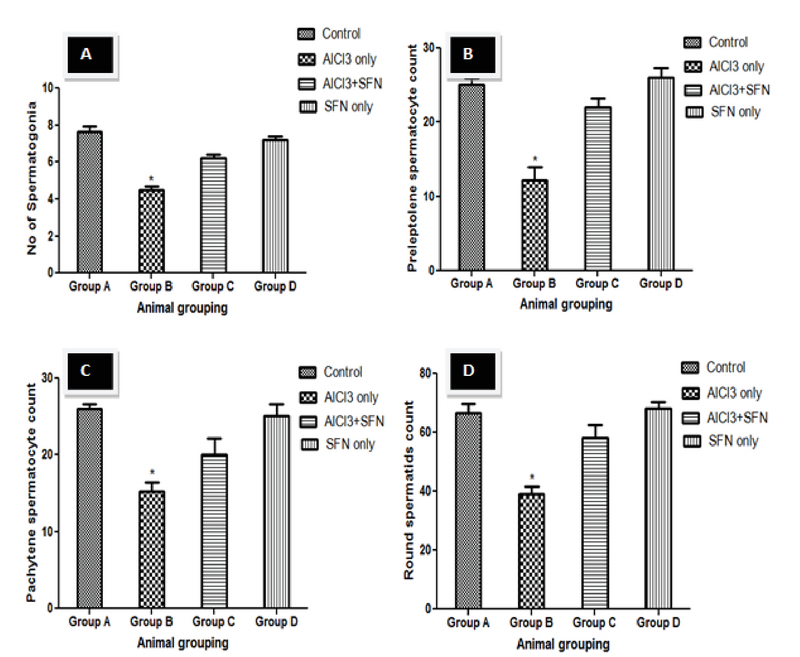
Effect of SFN on spermatogenic cell count on AlCl${}_{3}$ in normal and experimental rats (n = 8). *P < 0.05 compared with the control. Values expressed in Mean ± SEM.

The testicular germ cell count such as spermatogonia, preleptotene and pachytene spermatocytes, and round spermatids count in the seminiferous tubules showed a significant decrease in the counts compared to the control (p = 0.02; Figure 6 A-D). Although, the germ cell count after the administration of SFN and AlCl${}_{3}$ was significantly improved compared to the AlCl${}_{3}$ only group, but it was not statistically significant compared to the SFN only and control groups, respectively.

**Table 1:** Effect of SFN on testicular and body weights treated on AlCl${}_{3}$ in normal and experimental rats (n = 8)


**Parameters**	**Group A**	**Group B**	**Group C**	**Group D**
**Body weight (g)**	205 ± 4	174 ± 7*	200 ± 11	207 ± 12
**Testis weight (g)**	1.55 ± 0.02	0.9 ± 0.05*	1.35 ± 0.04	1.45 ± 0.06
*P < 0.02 compared with the control. Values expressed in Mean ± SEM				

## 4. Discussion

An emerging pandemic global public health issue after cancer and cardiovascular diseases is infertility due to increase in testicular cancer (35) and based on the analysis on semen parameters such as reduction in sperm counts and qualities in various countries (36, 37). The exposure of male individual to environmental toxicant is regarded as the channel that results in reduced sperm counts and infertility (1, 2). Aluminum is considered as the most common metallic element detectable in natural waters, animal, and plant tissues (3). Compounds of Al due to its reactivity with other elements such as Sulphur and chloride are widely used in many products such as storage utensils, household cookware, food additives, toothpaste,and pharmaceuticals (antacids, vaccines, allergy injections, and anti-diarrhea) (3). The enormous rate of exposure to Al increases the chances of health-related issues to human due to increase metallic concentration in various organs thereby damaging various tissue of the body including testicular tissues of animals and humans (38). Testicular weight is crucial in the evaluation of male fertility test due to its important association in sperm production (39). In our study, the decrease in body and testicular weights observed after AlCl${}_{3}$ only administration could be correlated to the deleterious effect of the toxicant on body metabolism and testicular architecture, thereby resulting in spermatogenesis disruption. Previous research also concur that Al intoxication causes drastic decrease in testicular weight resulting in germinal EE disruption and inadequate TT production (40, 41). However, SFN attenuated the body and testicular weight loss in combined administration of SFN and AlCl${}_{3}$ group thereby restoring the testicular function.

The seminal fluid analysis (sperm count, sperm motility, sperm viability) were significantly declined in the AlCl${}_{3}$ only group thereby causing oligospermia due to increased oxidative stress-induced damage and decreased concentration of scavenging enzymes (42, 43). Previous studies also showed similar decrease in sperm count and sperm motility after exposure to various environmental toxicants in different experimental animal models (44-46). However, the combined administration of SFN and AlCl${}_{3}$ increases the motility, concentration, and viability of the spermatozoa thereby mitigating the effects of AlCl${}_{3}$ intoxication on testicular tissue.

The process of spermatogenesis has been implicated to be under the regulation of reproductive hormones such as TT, FSH, and LH. In this present study, our results showed a decrease in this reproductive hormone after AlCl${}_{3}$ administration, thereby suggesting a decline in the role of anterior pituitary and Leydig cells. Previous studies have also observed that the decrease in the level of TT, FSH, and LH hormones in adult rats were due to several environmental agents (47-49). In addition, previous research deduced that the decrease in the level of TT synthesis could be due to the deleterious effects of testicular toxicant (such as NO, AlCl) on the Leydig cells and also the conversion of androsterone to TT due to decreased activity of 17-ketosteroid reductase enzyme (50, 51). However, the SFN and AlCl${}_{3}$ combined-treated group showed a significant improvement in serum FSH, LH, and TT levels that can be linked to the ameliorative potential of SFN on AlCl${}_{3}$ testicular toxicity in the release of gonadotrophin-releasing hormone (GnRH) secretion in the hypothalamus (52).

The antioxidant defense system prevents the cells of the body against the injurious effect of Reactive Oxygen Species (ROS) produced due to exposure to environmental toxicants, ultimately inducing toxicity to the reproductive system by perturbing the pro-oxidant and thereby leading to oxidative stress (53). Our study showed that exposure to AlCl${}_{3}$ decreased the antioxidant enzymes such as SOD, CAT, GSH and correspondingly increased the MDA level. The decline in the activities of the antioxidant enzymes observed in this study revealed that the antioxidant system was impaired, thereby inducing oxidative stress induced-testicular toxicity. Previous research have showed that the production of oxidative stress due to metallic exposure decrease enzyme defense mechanism, thereby causing spermatozoa cytotoxicity (54). In addition, the inhibition of sperm functions and male infertility was also reported to occur through toxicity of lipid peroxides via generation of reactive oxygen species (54, 55). However, the co-administration of SFN and AlCl${}_{3}$ in this study showed ameliorative effects against oxidative injury by increasing the levels of antioxidant enzymes (SOD, CAT, GSH) with corresponding decrease in lipid peroxidation. It could be deduced that SFN decrease the free radicals levels via its free radical scavenging activity, especially oxygen radicals, and modulates several cytokines release and activities of testicular enzymes.

The histomorphological features of the testis are critical and usually refer to as the endpoint in the evaluation of male fertility assessment and reproductive toxicity (56). In our study, histological observation of animals that received AlCl${}_{3}$ only showed various distortions such as shrinked seminiferous tubules, degeneration of Leydig cells, thinner germinal EE, spermatogenesis disruption, and absence of spermatozoa in the EL. Previous studies have also reported similar changes in the histoarchitecture of the testis after exposure to different environmental toxicant (49, 57). The alteration in testicular histomorphology by metallic toxicant might be due to oxidative stress, thereby causing distortion of the steroidogenic activity of the Leydig cells by penetration through the blood-testis barrier. However, the administration of SFN in AlCl${}_{3}$-induced testicular damage showed its protective ability on spermatogenesis and tubular atrophy that was confirmed in our study by the histomorphological observation that showed distinctive increase in seminiferous tubules diameter and presence of spermatozoa in the EL. Previous studies have showed that normal spermatogenesis is achievable in oxidative stress-induced testicular toxicity caused by environmental toxicant by several antioxidant-rich agents, thereby increasing the endocrine activity of the Leydig and Sertoli cells (58-60). Furthermore, the histomorphological observation in our study corroborates with the decrease in the number of spermatogonia, preleptotene, pachytene spermatocytes, and round spermatids in AlCl${}_{3}$ only group suggesting declined spermatogenic activity. The increased oxidative stress and lipid peroxidation could increase apoptosis of the germ cells. Previous studies also showed that apoptosis of the spermatogonia and primary spermatocyte can occur via microtubule targeting and mitotic arrest after exposure of environmental toxicant (61) and decreased diameter of the seminiferous tubule could also be an indicator of defective spermatogenesis (62, 63).

## 5. Conclusion

The present study revealed the ameliorative response of SFN on AlCl${}_{3}$-induced testicular toxicity on blood LH, FSH, and TT through oxidative stress. The protective function of SFN may preserve the functional integrity of the testis against environmental toxicity.

##  Conflict of Interest

The authors declare that there is no conflict of interests regarding the publication of this paper.
